# Fully Integrated Light-Sensing Stimulator Design for Subretinal Implants

**DOI:** 10.3390/s19030536

**Published:** 2019-01-28

**Authors:** Hosung Kang, Wajahat H. Abbasi, Seong-Woo Kim, Jungsuk Kim

**Affiliations:** 1 Department of Medical Science, Korea University, Seoul 02841, Korea; 2017010569@korea.ac.kr; 2 Department of Health Science and Technology, GAIHST, Incheon 21999, Korea; Waj@bme.gachon.ac.kr; 3 Department of Ophthalmology, Korea University Guro Hospital, Korea University College of Medicine, Seoul 08308, Korea; 4 Department of Biomedical Engineering, Gachon University, Incheon 21936, Korea

**Keywords:** subretinal prosthesis, photodiode, high-density pixels, ex-vivo demonstration, light sensor, digital controller, implantable device

## Abstract

This paper presents a fully integrated photodiode-based low-power and low-mismatch stimulator for a subretinal prosthesis. It is known that a subretinal prosthesis achieves 1600-pixel stimulators on a limited single-chip area that is implanted beneath the bipolar cell layer. However, the high-density pixels cause high power dissipation during stimulation and high fabrication costs because of special process technologies such as the complementary metal-oxide semiconductor CMOS image sensor process. In addition, the many residual charges arising from the high-density pixel stimulation have deleterious effects, such as tissue damage and electrode corrosion, on the retina tissue. In this work, we adopted a switched-capacitor current mirror technique for the single-pixel stimulator (SPStim) that enables low power consumption and low mismatch in the subretinal device. The customized P+/N-well photodiode used to sense the incident light in the SPStim also reduces the fabrication cost. The 64-pixel stimulators are fabricated in a standard 0.35-μm CMOS process along with a global digital controller, which occupies a chip area of 4.3 × 3.2 mm^2^ and are ex-vivo demonstrated using a dissected pig eyeball. According to measured results, the SPStim accomplishes a maximum biphasic pulse amplitude of 143 μA, which dissipates an average power of 167 μW in a stimulation period of 5 ms, and an average mismatch of 1.12 % between the cathodic and anodic pulses.

## 1. Introduction

Retinal implants offer great promise for restoring vision to patients who suffer from retinal diseases such as retina pigmentosa and age-related macular degeneration. According to the anatomical position, retinal prostheses can be classified as epiretinal [1,2], subretinal [3,4,5], or suprachoroidal [6,7] devices. Among them, it has been reported that the subretinal implant can provide high pixel density of up to 1600 pixels [8], on a limited silicon chip area [9]; this is possible because the stimulator in the subretinal device does not need a high-resolution current-steering digital-to-analog converter (DAC) and its own local digital controller, which are employed to generate various biphasic current pulses for epiretinal and suprachoroidal prosthetics. The high-resolution DAC and the digital controller occupy a large area in a single-pixel stimulator (SPStim); the area becomes dominant with high-voltage CMOS (complementary metal-oxide semiconductor) process technology [2].

**Figure 1:**
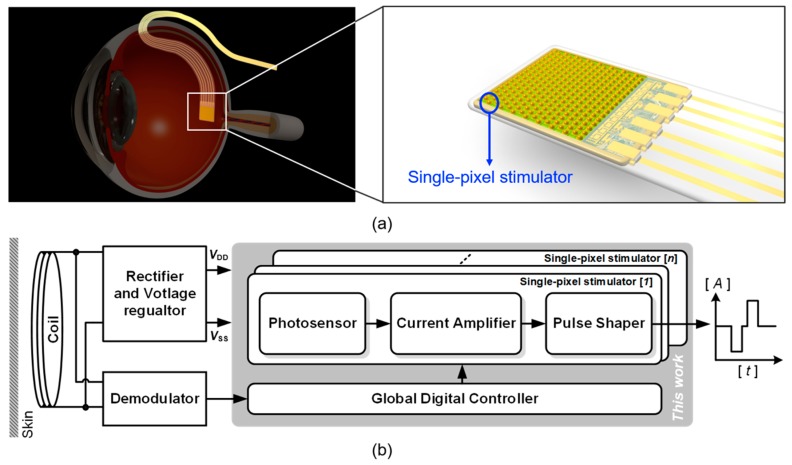
(**a**) System overview of a subretinal prosthesis that is implanted beneath the bipolar cell layer, (**b**) simplified architecture of the subretinal device including the single-pixel stimulator.

Figure 1a shows a subretinal prosthesis that is inserted into the subretinal space of the eye via ab-externo approach. The prosthetic chip and cable, which works to deliver a power and a command data from an inductive coupling coil located in the retroauricular area, enters the subretinal space passing through the partial-thickness scleral flap. Then, the rest cable outside the eye is buried under the temporalis muscle. Figure 1b depicts the general architecture of a single-pixel stimulator (SPStim) for a subretinal implant, which is composed of a photosensor, a current amplifier, and a pulse shaper. A photodiode in the photosensor produces a dark current corresponding to the light intensity incoming onto the retina. The dark current is in the order of nano-amperes and has a monophasic shape. The current amplifier in Figure 1b works to amplify the dark current from tens to hundreds of microamperes. Finally, the pulse shaper reshapes the amplified monophasic signal to a biphasic current pulse, which consists of a rectangular cathodic and anodic pulse, and then delivers the biphasic signal into the bipolar cells interfaced with a microelectrode.

The single-pixel stimulator for the subretinal prosthesis should meet three design requirements as follows. First, the amplitude mismatch of the cathodic and anodic pulses should be as low as possible in order to reduce the residual charge on the retina tissue after stimulation. The residual charge sometimes induces unwanted spike excitation that results in tissue damage, as well as electrode corrosion that impedes the delivery of stimulation charge to the tissue from the stimulator output [10]. Thus, it is indispensable to minimize the amplitude mismatch of the biphasic stimulus current. Second, it must be fully controllable by digital logic embedded on the same chip in order to generate the diverse shapes of the biphasic stimulus current. Because visually impaired patients have individually different thresholds for exciting the nerves in the retina [11], after implantation, the stimulus current shapes must be adjusted, including the amplitude, width, period of the biphasic stimuli, and the interphase delay between the cathodic and anodic pulses. Finally, the stimulator necessitates a wide dynamic output range to produce various amplitudes of the biphasic current owing to the different thresholds of the patients to excite their retinal nerves. A simple way to widen the output dynamics is to raise the power supply rail. However, the high-voltage operation leads to high power consumption in the SPStim, which would become worse in a subretinal device with high-density pixels. We thus must devise a new SPStim architecture to accomplish the wide dynamic output range while maintaining low-voltage operation.

Over the last decade, a few single-pixel stimulators have been presented for subretinal implants to implement high-density stimulator arrays [3], low-voltage operation [12], and low mismatch [6,13]. To the authors’ best knowledge, however, any results that meet all of these design requirements have not yet been presented. Thus motivated, in this work we propose a SPStim design that adopts a switched-capacitor current mirror technique that achieves both low mismatch and wide dynamic output range in low-voltage operation. This stimulator circuit was fabricated in a standard 0.35-μm 4M2P CMOS process and ex-vivo demonstrated employing a dissected pig eyeball. 

## 2. Methods

**Figure 2:**
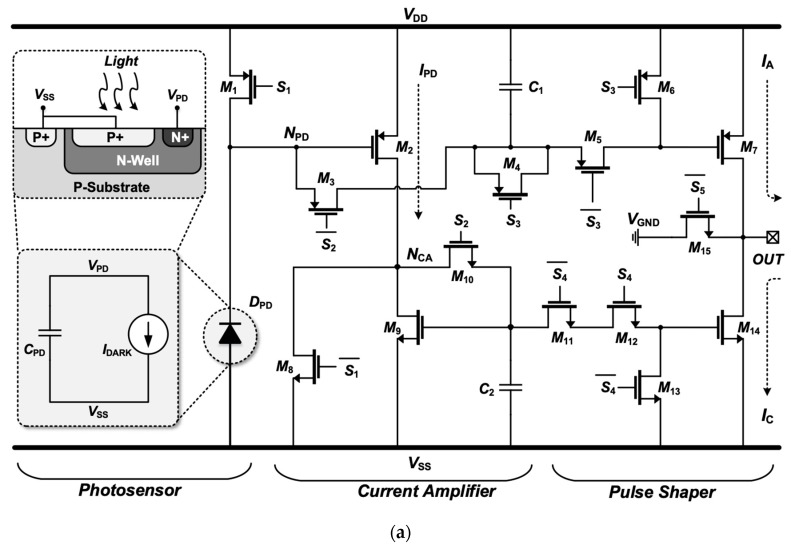

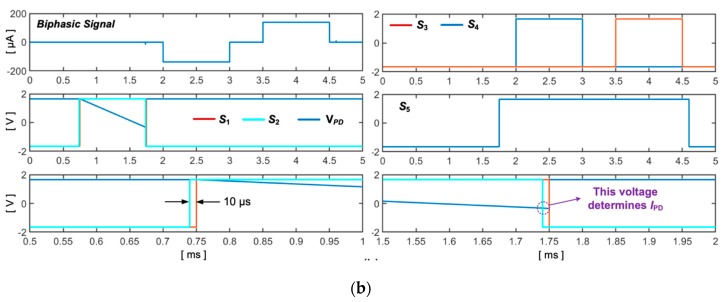
(**a**) Circuit diagram of the proposed single-pixel stimulator adopting a customized photodiode and a switched capacitor current mirror technique; (**b**) simulated transient waveform to show input and analog output.

Figure 2 shows the circuit diagram of the proposed SPStim, which is composed of three stages: (1) a photosensor, (2) a current amplifier, and (3) a pulse shaper, as illustrated in Figure 1b. Also shown are simulated transient waveforms for the SPStim’s digital inputs and analog output. The photosensor stage is composed of a photodiode D_PD_ and a PMOS transistor M_1_, which works as a switch to reset D_PD_. In this design, a customized photodiode that has the structure of a P+ and N-well as illustrated in Figure 2a has been employed because of its high sensitivity [14] and relatively low fabrication cost compared with the CMOS image sensor process that requires a deeper epitaxial layer, anti-reflective coating and optimization of passivation in order to minimize interference [15]. D_PD_ can be modeled as a dark current source I_DARK_, which is proportional to the incident light intensity, and a parasitic capacitor C_PD_, which arises from the junction area of the P+ and N-well in the diode, C_gd1_, C_gs3_, C_gs2_, and C_gs2_. Here, C_gd_, and C_gs_ denote a gate-drain and gate-source parasitic capacitance of the transistors. Although I_DARK_ and C_PD_ respectively vary with the light intensity and the dimension of D_PD_ and its adjacent transistors, we set I_DARK_ and C_PD_ to 8 nA and 6 pF for the simulation environment.

The proposed SPStim operates in four different modes: the preset mode (0–0.74 ms in Figure 2b), reset mode (0.74–0.75 ms), charging mode (0.75–1.75 ms), and stimulation mode (0.175–4.6 ms), followed by the next preset mode (0.46–5 ms). During the preset mode, where all signals of the digital controller are set to logic 0, the PMOS transistor M_1_ is fully turned on, and as a result, C_PD_ is charged up to V_DD_. The stages other than the photodiode stage are deactivated in this mode to diminish unnecessary power dissipation. By applying a logic 1 to the S_2_ on M_3_ and M_10_ in the current amplifier stage, the reset mode begins, where the capacitors C_1_ and C_2_ are reset to V_DD_ through M_1_ and M_3_, and to V_SS_ through M_8_ and M_10_, respectively. Thus, the previous stimulation memory recorded on C_1_ and C_2_ is erased so as to receive new stimulation information.

The charging mode starts when the S_1_ on M_1_ and M_8_ becomes logic 1. The S_1_ is synchronized with the S_2_, but its rising and falling edges lag those of the S_2_ by 10 μs. We generate the S_1_ from the S_2_ using a simple delay chain as presented in [16]. As the S_1_ turns high, the transistor M_1_ is immediately disabled. Because of the I_DARK_, the node voltage of N_PD_, here called V_PD_, begins to steadily drop from V_DD_ to V_DD_ − I_DARK_ × ΔT/(C_1_ + C_PD_), where ΔT indicates the duration of the charging mode (0.75–1.75 ms in this simulation). The drop in V_PD_ enables the PMOS transistor M_2_, thereby entering it to a saturation region. Accordingly, the current I_PD_ generated from M_2_ can be defined as

(1) $$I_{\mathrm{PD}}=\frac{1}{2}\mu_{P}C_{\mathrm{OX}}{\left(\frac{W}{L}\right)}_{2}{\left(V_{\mathrm{DD}}-V_{\mathrm{PD}}-\left|V_{\mathrm{TH},P}\right|\right)}^{2}=\frac{1}{2}\mu_{P}C_{\mathrm{OX}}{\left(\frac{W}{L}\right)}_{2}{\left(\frac{I_{\mathrm{DARK}}\times \Delta T}{C_{1}+C_{\mathrm{PD}}}-\left|V_{\mathrm{TH},P}\right|\right)}^{2}$$

Here, *μ*_P_, *C*_OX_, *W/L*, and *V*_TH,P_ are the channel mobility, gate oxide capacitance per unit area, aspect ratio, and PMOS threshold voltage, respectively. This equation shows that the small current *I*_DARK_ in the nano-ampere range is amplified to a stimulus current in the tens to hundreds of micro-amperes by adjusting the charging duration Δ*T*. In this work, we aimed to generate a stimulation current of 150 μA at maximum. Because *I*_PD_ produced from M_2_ flows out to *V*_SS_ only through M_9_, *I*_PD_ can be transformed to:

(2) $$I_{\mathrm{PD}}=\frac{1}{2}\mu_{P}C_{\mathrm{OX}}{\left(\frac{W}{L}\right)}_{2}{\left(V_{\mathrm{DD}}-V_{\mathrm{PD}}-\left|V_{\mathrm{TH},P}\right|\right)}^{2}=\frac{1}{2}\mu_{N}C_{\mathrm{OX}}{\left(\frac{W}{L}\right)}_{9}{\left(V_{\mathrm{CA}}-V_{\mathrm{TH},N}\right)}^{2}$$

Here, *μ*_N_ and *V*_CA_ denote the channel mobility of NMOS transistor and the node voltage of *N*_CA_, respectively. *V*_CA_ can be rewritten as:

(3) $$V_{\mathrm{CA}}=\sqrt{\frac{2I_{\mathrm{PD}}}{\mu_{N}C_{\mathrm{OX}}{\left(\frac{W}{L}\right)}_{9}}}+V_{\mathrm{TH},N}$$

Including *I*_PD_, *V*_CA_ changes with the incident light intensity and Δ*T*. Consequently, the voltages *V*_PD_ and *V*_CA_ are correlated with *I*_PD_, which is used as the stimulus current, and are recorded on C_1_ and C_2_ at 1.74 ms in Figure 2b when the S_2_ turns back to a logic 0. After 10 μs, the S_1_ turns off again. Here, the 10-μs delay reduces the deleterious effect of charge injection that can lead to voltage fluctuations on C_1_ and C_2_ by the S_1_ switching.

The stimulation mode starts when the S_5_ signal turns to a logic 1 at 1.75 ms in Figure 2b. In other modes except for this stimulation one, the PMOS transistor M_15_ always stays “on” to prevent DC current from being leaked out to the retina tissue. By turning the S_4_ to a logic 1, the *V*_CA_ stored on C_2_ is applied to the gate of the NMOS transistor M_14_, thereby generating a cathodic current *I*_C_:

(4) IC=12μNCOX(WL)14(VCA−VTH,N)2 and thus ICIPD=(WL)14(WL)9

Here, the channel-length modulation effect can be reduced by enlarging the length of M_14_ while still making the aspect ratio (W/L)_14_ equal to (W/L)_9_. In order to generate an anodic current pulse, the conventional designs exploit an additional current-mirror branch [4,6,12,13]. This results in unnecessary power consumption and a large mismatch between the cathodic and anodic currents.

Under the assumption that the additional current-mirror branch to copy *I*_PD_ for *I*_A_ exits in Figure 2a, the average power that the SPStim dissipates can be approximated to:

(5) $$P_{\mathrm{av}}\approx \underset{\mathrm{Current}\;\mathrm{Amplifier}}{\underset{︸}{\frac{1}{T_{S}}\int_{0}^{T_{S}}I_{\mathrm{PD}}\times \left(V_{\mathrm{DD}}-V_{\mathrm{SS}}\right)\mathrm{dt}}}+\underset{\mathrm{Pulse}\;\mathrm{Shaper}}{\underset{︸}{\frac{1}{T_{S}}\int_{0}^{T_{S}}\left[I_{A}\times V_{\mathrm{DD}}-I_{C}\times V_{\mathrm{SS}}+I_{C}\times \left(V_{\mathrm{DD}}-V_{\mathrm{SS}}\right)\right]\mathrm{dt}}}$$

Here, *I*_DARK_ is ignored because of *I*_PD_ >> *I*_DARK_ and *T*_S_ denotes the period of the biphasic waveform. In the second term of Equation (5), *I*_C_ × (*V*_DD_ − *V*_SS_) arises from the additional branch. Thus, the average power dissipated by all mirroring branches can be written as:

(6) $$P_{\mathrm{tot},\mathrm{branch}}\approx N\times \frac{1}{T_{S}}\int_{0}^{T_{S}}I_{C}\times \left(V_{\mathrm{DD}}-V_{\mathrm{SS}}\right)\mathrm{dt}$$

where *N* means the number of pixels simultaneously stimulated. N also becomes higher in a subretinal device with high-density pixels. 

By adopting the capacitor C_1_ to directly record the value of *V*_PD_, in this work, we remove the need for the additional branch, thus reducing both mismatch and power dissipation. As a result, the average power of the SPStim can be approximately expressed as:

(7) $$P_{\mathrm{av}}\approx \underset{\mathrm{Current}\;\mathrm{Amplifier}}{\underset{︸}{\frac{1}{T_{S}}\int_{0}^{T_{S}}I_{\mathrm{PD}}\times \left(V_{\mathrm{DD}}-V_{\mathrm{SS}}\right)\mathrm{dt}}}+\underset{\mathrm{Pulse}\;\mathrm{shaper}}{\underset{︸}{\frac{1}{T_{S}}\int_{0}^{T_{S}}\left(I_{A}\times V_{\mathrm{DD}}-I_{C}\times V_{\mathrm{SS}}\right)\mathrm{dt}}}$$

This equation shows that the proposed SPStim consumes relatively less power compared to a stimulator using the addition current-mirror branch.

After the cathodic pulse, a logic 1 is applied to the PMOS transistor M_5_, and then the *V*_PD_ produces the anodic current *I*_A_ from M_7_. The dummy transistors M_4_ and M_11_ are also harnessed to avoid charge injection caused by the M_5_ and M_12_ switching. Consequently, the two switched-capacitor current mirrors adopted in the proposed SPStim make it possible to reduce power consumption in high-density pixel array; reduce the mismatch, which allows low residual charge on the tissue after stimulation; and widen the output dynamic range, which enables low-voltage operation. 

## 3. Measurement

**Figure 3:**
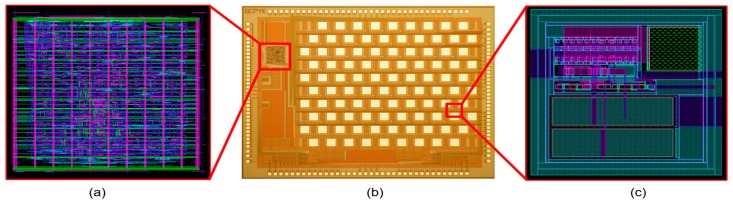
(**a**) Layout of the global digital controller embedded onto the signal chip with a 64-pixel stimulator array (its active area is gauged as 441.5 × 425.5 μm^2^); (**b**) micrograph of the 64-pixel stimulator array; (**c**) layout of the proposed single-pixel stimulator, which occupies an active area of 114 × 117 μm^2.^

**Figure 4:**
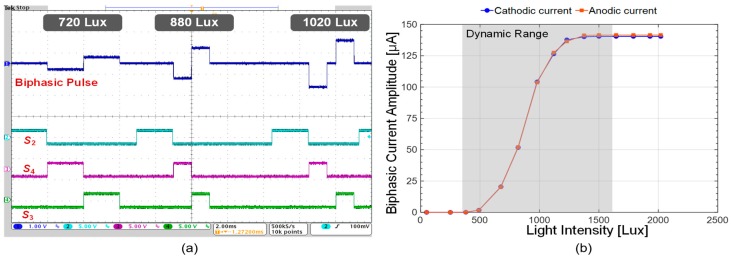
(**a**) Measured transient waveforms for the biphasic current and digital input pulses, where a 10-kΩ resistor was used to model the electrode resistance; (**b**) anodic and cathodic current amplitudes varying with incident light intensity.

Figure 3 shows a micrograph of the 64-pixel stimulator array adopting the proposed architecture, and the global digital controller to produce digital signals for S_1_, S_2_, S_3_, S_4_ and S_5_ in Figure 2a; both were fabricated in a single chip using a standard 0.35-µm 4M2P CMOS process. This full chip occupies an active area of 4.3 × 3.2 mm^2^. We first conducted a benchtop experiment to measure the transient bip-hasic current waveforms, which vary with the incident light intensity, using a light source (Model: Newport 66088-LED). Figure 4a illustrates the biphasic current waveform and its digital control signals observed with an oscilloscope (Model: Tektronix MSO4104). By changing the light intensity and supplying various digital pulses to the SPStim, we successfully generated diverse biphasic current shapes in amplitude, width, interphase and period. In this benchtop experiment, we used a 10-kΩ resistor to simply mimic the electrode resistance and set the time width for S_2_ to 2 ms for fair comparison of the light-dependent biphasic current amplitudes. 

Figure 4b shows the measured anodic (red line) and cathodic (blue line) amplitudes relying on the incident light intensity. 

The observed results show that the biphasic stimulus current of the proposed SPStim varies from 0 to 143 μA in the dynamic range from 400 to 1600 lux, where an average mismatch of 1.12% was calculated using the following equation.

(8) $$\mathrm{Mismatch}=\frac{1}{N}\sum_{n=1}^{N}\frac{\left|A_{\mathrm{anodic}}\left(n\right)-A_{\mathrm{cathodic}}\left(n\right)\right|}{\max\left[A_{\mathrm{anodic}}\left(n\right){,}^{}A_{\mathrm{cathodic}}\left(n\right)\right]}$$

Here, *N*, *A*_anodic_ and *A*_cathodic_ indicate the number of measured samples, the anodic and cathodic pulse amplitudes in the dynamic range. This low mismatch is due to the switched-capacitor current mirror technique described in Figure 2a. The charge remaining on the tissue after stimulation was completely removed by turning on the charge-balancing switch M_15_.

**Figure 5:**
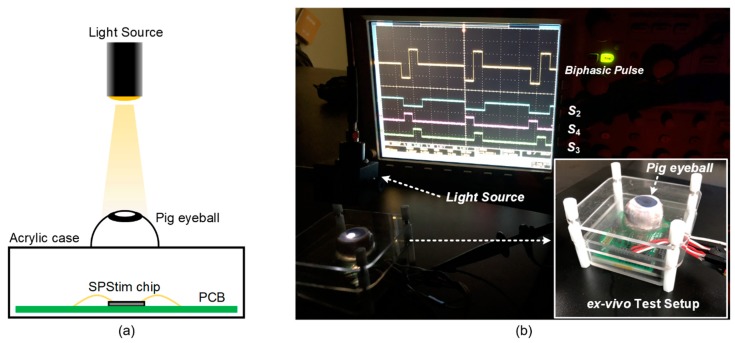
(**a**) Illustration of the ex-vivo demonstration setup; (**b**) ex-vivo experiment using a dissected pig eyeball.

Next, we ex-vivo demonstrated the proposed SPStim using a dissected pig eyeball as shown in Figure 5. Here, it is observed that the biphasic pulse amplitudes are reduced by approximately 29% compared to the waveforms measured in Figure 4a. This is because the incident light intensity is attenuated when passing through the pig eyeball lens [17]. In terms of Equation (1), the attenuated light reduces I_DARK_ in the photodiode, thereby reducing the stimulation current I_PD_. To compensate for the reduced I_PD_, we elongated the S_2_ (ΔT in Equation (1)) from 2 ms (see Figure 4a) to 2.5 ms, and as a result, the SP-Stim could generate the same biphasic pulse amplitudes as observed in Figure 4b in the same dynamic range. This demonstration shows that the customized global digital controller is necessary to adjust biphasic stimulus pulses after implantation. Finally, the overall performance of the proposed SPStim is summarized in Table 1.

## 4. Conclusions

A fully integrated low-power and low-mismatch SPStim was designed for subretinal implants. By adopting two switched-capacitor current mirrors for anodic and cathodic pulse generation, in this work, we achieved relatively less power consumption, compared to a SPStim using the additional current-mirror branch to generate an anodic or a cathodic stimulation pulses, and low mismatch to decrease the residual charges that can have deleterious effects on the retina. Using the customized P+/N-well photodiode in a standard CMOS process also enabled relatively lower fabrication cost than a complex CMOS images sensor process. The 64-pixel stimulators employing the proposed architecture were fabricated in a standard 0.35-μm CMOS process and ex-vivo tested with a dissected pig eyeball. From the measured results, the device achieves a maximum biphasic pulse amplitude of 143 μA, an output dynamic range of 86.7%, and a mismatch of 1.12% on average between the anodic and cathodic pulses. The customized global digital controller, for producing diverse biphasic stimulus currents in amplitude, width, interphase delay, and period, was embedded on the same chip with the 64-pixel stimulators and used to compensate for the attenuated light intensity passing through the pig eyeball lens in ex-vivo experiments. In future work, a refined 64-pixel stimulator array will functionalize the wireless power and data telemetry that we are developing now. 

## Figures and Tables

**Table 1:** Electrical performance Summary and comparison of the proposed single-pixel stimulator (SPStim).

	[18]	[6]	[12]	[13]	[3,18,19,20]	This Work
CMOS Process	0.35-μm standard	0.18-μm CIS **	0.18-μm CIS **	0.35-μm BCD ***	0.35-μm CIS **	0.35-μm standard
Supply voltage [V]	5	± 1.65	0.5, 1.8	5, 12	± 2	± 1.65
Chip area [mm^2^]	1.0 × 2.7	2.2 × 2.2	1.9 × 1.9	2.5 × 1.2	3 × 3.5	4.3 × 3.2
Single pixel area [μm^2^]	200 × 200	127 × 167	30 × 30	55 × 50	30 × 30	114 × 117
Dynamic range [Lux]	-	400–1200	1–1000	100–250	0.1–10,000	400–1600
Pulse amplitude * [μA]	50–1050	0–343	0–50	0–300	0–100	0–143
On-chip digital controller	Yes	No	Yes	No	Yes	Yes
Pulse width * [ms]	-	-	-	-	4	0.5–5
Pulse period * [ms]	-	-	60	-	3–100	1–50
Interphase delay * [ms]	-	-	-	-	0 ms	0–2 ms
Mismatch [%]	-	4.85	-	-	-	1.12
Stimulation method	Charge-balanced biphasic current stimulation					

* Pulse: Biphasic Pulse, ** CIS: CMOS Image Sensor, *** BCD: Bipolar CMOS and Double diffused MOS(DMOS).
